# Structural Alteration of the Dorsal Visual Network in DLB Patients with Visual Hallucinations: A Cortical Thickness MRI Study

**DOI:** 10.1371/journal.pone.0086624

**Published:** 2014-01-23

**Authors:** Stefano Delli Pizzi, Raffaella Franciotti, Armando Tartaro, Massimo Caulo, Astrid Thomas, Marco Onofrj, Laura Bonanni

**Affiliations:** 1 Department of Neuroscience and Imaging, “G. d’Annunzio” University, Chieti, Italy; 2 Aging Research Centre, Ce.S.I., “G. d’Annunzio” University Foundation, Chieti, Italy; 3 Institute for Advanced Biomedical Technologies (ITAB), “G. d’Annunzio” University Foundation, Chieti, Italy; Banner Alzheimer’s Institute, United States of America

## Abstract

Visual hallucinations (VH) represent one of the core features in discriminating dementia with Lewy bodies (DLB) from Alzheimer’s Disease (AD). Previous studies reported that in DLB patients functional alterations of the parieto-occipital regions were correlated with the presence of VH. The aim of our study was to assess whether morphological changes in specific cortical regions of DLB could be related to the presence and severity of VH. We performed a cortical thickness analysis on magnetic resonance imaging data in a cohort including 18 DLB patients, 15 AD patients and 14 healthy control subjects. Relatively to DLB group, correlation analysis between the cortical thickness and the Neuropsychiatric Inventory (NPI) hallucination item scores was also performed. Cortical thickness was reduced bilaterally in DLB compared to controls in the pericalcarine and lingual gyri, cuneus, precuneus, superior parietal gyrus. Cortical thinning was found bilaterally in AD compared to controls in temporal cortex including the superior and middle temporal gyrus, part of inferior temporal cortex, temporal pole and insula. Inferior parietal and supramarginal gyri were also affected bilaterally in AD as compared to controls. The comparison between DLB and AD evidenced cortical thinning in DLB group in the right posterior regions including superior parietal gyrus, precuneus, cuneus, pericalcarine and lingual gyri. Furthermore, the correlation analysis between cortical thickness and NPI hallucination item scores showed that the structural alteration in the dorsal visual regions including superior parietal gyrus and precuneus closely correlated with the occurrence and severity of VH. We suggest that structural changes in key regions of the dorsal visual network may play a crucial role in the physiopathology of VH in DLB patients.

## Introduction

Dementia with Lewy bodies (DLB) is the second most common form of dementia in the elderly after Alzheimer’s Disease (AD). Despite the precise nosological relationship between DLB and AD remains uncertain, persistent visual hallucinations (VH) along with fluctuations in cognitive function and parkinsonism represent the core feature in discriminating DLB from AD and other dementias [1].

The physiopathology underlying VH in DLB is not well understood. Causative models for complex VH have suggested that the origin of this deficit may be related to a range of pathological alterations affecting both the dorsal visual stream, specialized for visuo-spatial attention and location, and the ventral visual stream, designated to object recognition [2]. Abnormalities within the ventral visual stream, revealed by white matter damage to the inferior longitudinal fasciculus [3] and by increased Lewy body pathology in the temporal lobe [4], [5], were reported in DLB patients with VH compared to those without VH, supporting the role of a bottom-up dysfunction. Conversely, increased atrophy [6] and Lewy body pathology [5] within frontal lobes were observed in hallucinating DLB in comparison with non-hallucinating DLB patients, supporting a top-down mechanism in the genesis of VH.

In this context, structural Magnetic Resonance Imaging (MRI) could be a powerful tool to investigate the aetiology of core symptoms in DLB. To date, several studies by using voxel-based morphometry (VBM) found widespread cortical sites of grey matter atrophy in DLB patients compared to healthy controls [7]–[9] but only few of these studies could directly relate structural evidences to the appearance of VH [6]. However, VBM presents some limitations: the tissue volume changes among groups are measured on a voxel by voxel basis after brain normalization to a standardized space. The interpretation of results can be difficult because the algorithm of analysis can involve brain sites that are in close spatial proximity but not closely anatomically connected, and given that any physical characteristic is measured directly [10].

Cortical thickness approach allows to perform cortical parcellation and to measure with great reliability the pathological thinning of the cortical grey matter. By measuring a real physical quantity that is much easier to interpret and to localize in comparison to the three-dimensional VBM, cortical thickness methods provide a better localization of structural changes because the smoothing procedure is performed on a two-dimensional manifold which reduces morphological artefacts due to ageing and pathological processes [11].

Despite the potential advantage of cortical thickness analysis, to date this approach has not been applied to DLB patients.

In the present study, we performed cortical thickness analysis to verify the presence of cortical grey matter damage related to VH in DLB patients. To this aim, firstly, we compared DLB patients respect to healthy controls. Subsequently, we included a comparison against AD groups to exclude possible pathologic alterations arising as a result of a dementia process per se and not specific to DLB pathology. Finally, relatively to DLB group, we performed a correlation analysis between the cortical thickness and the Neuropsychiatric Inventory (NPI) hallucination item scores to highlight the cortical regions which were effectively related to the occurrence and severity of VH.

## Materials and Methods

### Study Sample

The study was approved by the Institutional and Ethics Committee of the University “G. d’Annunzio” Chieti-Pescara (ID#157801). All procedures were conducted according to the Declaration of Helsinki and subsequent revisions [12]. Data will be made freely available upon request. All patients (or their caregivers) and healthy subjects signed a written informed consent. 16 patients with AD and 19 patients with DLB were recruited from our case cohorts of patients referring to Memory and Movement Disorder Clinic; 15 age-matched healthy volunteers were recruited from our non-demented case register cohorts. The diagnosis of probable AD was based on National Institute of Neurological and Communicative Diseases and Stroke/Alzheimer’s Disease and Related Disorders Association criteria [13]. The diagnosis of probable DLB was made by consensus guidelines [1] with a specific restriction: we included only patients with the presence of complex, recurrent VH, plus at least one additional core feature (parkinsonism or cognitive fluctuations) or one core and one suggestive feature [1].

All patients underwent MRI scan within six months before the inclusion in the study and dopaminergic presynaptic ligand ioflupane SPECT (DAT scan).

### Clinical Assessment

Global tests of cognition included Clinical Dementia Rating (CDR), Mini Mental State Examination (MMSE), Dementia Rating scale-2 (DRS-2) [14] and Frontal Assessment Battery (FAB) [15]. The motor part of the Unified Parkinson’s Disease Rating scale (UPDRS) [16] was carried out to assess the presence and severity of parkinsonian signs. NPI was used to assess neuropsychiatric symptoms [17]; the occurrence and severity of VH was assessed by the NPI item-2. Minimal International Classification of Sleep Disorders criteria were performed to assess REM sleep Behaviour Disorder (RBD) [18]; Clinician Assessment of Fluctuations (CAF) [19], was used to evaluate the presence and severity of cognitive fluctuations. All patients were also assessed with electroencephalogram (EEG) recordings as EEG abnormalities characterised by parieto-occipital dominant frequency modifications has previously demonstrated to reliably differentiate probable DLB from AD patients [20].

Exclusion criteria were uncontrolled hypertension, myocardial ischemia, peripheral vascular disease and chronic kidney.

### MR Data Acquisition and Analysis

All acquisitions were carried out with a Philips Achieva 3 T scanner (Philips Medical System, Best, the Netherlands) equipped with 8-channel receiver coil. After scout and reference sequences, a 3-dimensional T_1_-Weighted Turbo Field-Echo sequence (3D T_1_−W TFE, TR/TE = 11/5 ms, slice thickness of 0.8 mm) was carried out. Participants with images characterized by motion artefacts were excluded from the analysis.

Cortical thickness on T_1_-weighted image was estimated at each vertex across the brain surface using Freesurfer software package (http://surfer.nmr.mgh.harvard.edu). This method has been previously described in detail by Fischl and Dale [21]. Specifically, cortical reconstruction process included magnetic field inhomogeneity correction, affine-registration to Talairach-atlas and skull-strip, segmentation of the subcortical white matter and deep grey matter volumetric structures, intensity normalization, tessellation of the grey and white matter boundary, automated topology correction, and surface deformation following intensity gradients to optimally place the grey and white matter and grey/cerebrospinal fluid borders at the location where the greatest shift in intensity defines the transition to the other tissue class. Once the cortical reconstruction process is complete, registration to a spherical atlas to match cortical geometry across subjects, and subsequent parcellation of the cerebral cortex into units based on gyral and sulcal structure were performed. Cortical thickness measurements were obtained by reconstructing representations of the gray/white matter boundary and the cortical surface. The distance between these two surfaces was calculated individually at each point across the cortical mantle: for each point on the white matter surface, the shortest distance to the pial surface was first computed; next, for each point on the pial surface, the shortest distance to the white matter was found, and the cortical thickness at that location was set to the average of these two values. All subjects were aligned to a common surface template using a high-resolution surface-based averaging technique that aligns cortical folding patterns. The spatial cortical thickness distribution was smoothed with a circularly symmetric Gaussian kernel of 10 mm.

Cortical brain regions affected by cortical thinning were classified by using the Desikan-Killiany Atlas integrated in FreeSurfer.

### Statistical Analysis

SPSS version 14.0 was used for statistical analysis. Analysis of variance (ANOVA) among groups and Tukey’s HSD post-hoc test was performed on demographic and clinical data. Chi-squared test was carried out for gender.

For each hemisphere, comparison among groups was performed by using general linear model (GLM), including cortical thickness as dependent factor and group (controls, AD and DLB) as independent factor. MMSE, CAF and UPDRS scores were included as covariates.

FreeSurfer processing stream was used to assess the pairwise differences among groups (https://surfer.nmr.mgh.harvard.edu/fswiki/FsgdFormat).

In DLB group, QDEC (Query, Design, Estimate, Contrast, https://surfer.nmr.mgh.harvard.edu/fswiki/FsTutorial/QdecGroupAnalysis_freeview) was used to individuate statistically significant regions where NPI hallucination item scores and thickness were correlated. MMSE, CAF, UPDRS scores were included as nuisance factors.

All results were corrected for multiple comparisons by using a pre-cached cluster-wise Monte-Carlo Simulation [22] and mapped on the surface. Significance level was set at p<0.05.

To perform partial correlations between NPI hallucination item scores and mean cortical thickness values in the posterior regions which were affected significantly in DLB in comparison with AD, the mean cortical thickness values were extracted from each region of interest (defined by Desikan-Killiany Atlas) by using “aparcstats2table” command line, (http://surfer.nmr.mgh.harvard.edu/fswiki/aparcstats2table). Next, SPSS version 14.0 was used for statistical computation and MMSE, CAF and UPDRS scores were included as nuisance factors.

## Results

One DLB patient, 1 AD patients and 1 control subject were excluded from the statistical analysis due to the presence of motion artefacts on MRI, leaving a total study cohort of 18 DLB, 15 AD and 14 control subjects.

### Demographic and Clinical Features

No differences on age, gender and educational level were found in the three groups (**table 1**). Dopamine-transporter hypocaptation in the caudate nuclei at SPECT-DAT scan was observed in all DLB patients, and it was bilateral in 12 patients. SPECT-DAT scan abnormalities were not observed in AD patients. Patients were treated with L-Dopa (all DLB patients), rivastigmine or donepezil (all AD and DLB patients, with no differences in daily dosages), quetiapine (9 DLB and 7 AD), clozapine (5 DLB), risperidone (5 AD) and clonazepam (the 16 DLB patients with RBD).

**Table 1:** Demographic and clinical features in all groups.

Characteristics	Controls (n = 14)	AD (n = 15)	DLB (n = 18)
Agea,b	75.5±5.3	75.6±7.6	75.4±4.0
Male gender (in percentage)^c^	50%	47%	50%
Disease duration (years)^d^	–	3.1±0.6	2.9±0.6
Education level (years)a,e	7±5	7±3	7±4
MMSEa,f	28.1±1.6	18.3±4.1	19.1±2.6
FABa,g	17.6±0.5	8.7±2.9	9.0±2.8
DRS-2a,h	136.6±0.6	91.7±18.3	92.8±13.8
CDRa,i	–	2.5±0.5	2.4±0.5
CAF	0.0±0.0	0.0±0.0	5.1±3.9
UPDRS III	0.0±0.0	0.0±0.0	24.7±6.7
NPI-item 2 hallucinations	0.0±0.0	0.0±0.0	5.0±3.2
VH (n. of patients affected)	–	–	18
RBD (n. of patients affected)	–	–	16
qEEG abnormalites (n. of patients affected)	–	–	18

Values are expressed as mean ± standard deviation (SD);

a the p-values were calculated using the one-way ANOVA; Tukey’s HSD *post-hoc* test was also perfomed when F-test was significant;

b main interaction among groups: F = 0.007, df = (2,47), p = 0.993;

c the p-values were calculated using chi-squared test: χ^2^ = 0.553, df = 2, p = 0.758;

d the p-values were calculated using the independent-samples t-test: t = −0.565, df = 31, p = 0.576;

e F = 0.007, df = (2,46), p = 0.762;

f main interaction among groups: F = 2581.393, df = (2,47), p<0.001; *post-hoc*: controls vs. AD, p<0.001; controls vs. DLB, p<0.001 and AD vs. DLB, p = 0.762;

g main interaction among groups: F = 1116.212, df = (2,47), p<0.001; *post-hoc*: controls vs. AD, p<0.001; controls vs. DLB, p<0.001 and AD vs. DLB, p = 0.917;

h main interaction among groups: F = 2956.939, df = (2,47), p<; *post-hoc*: controls vs. AD, p<; controls vs. DLB, p< and AD vs. DLB, p = 0.967;

i the p-values were calculated using the independent-samples t-test: t = −0.813, df = 31, p = 0.423.

Abbreviations: AD = Alzheimer’s Disease; CAF = Clinician Assessment of Fluctuations; DLB = dementia with Lewy bodies; DRS = Dementia Rating Scale; FAB = Frontal Assessment Battery; MMSE = Mini Mental State Examination; N/A = not applicable; NPI = Neuropsychiatric Inventory; RBD = REM Sleep Behaviour Disorder; qEEG = quantitative electroencephalogram; UPDRS = Unified Parkinson’s Disease Rating Scale; VH = visual hallucinations.

Neuropsychological test scores for each group are presented in **table 1**. AD and DLB patients exhibited no differences on global test of cognition (CDR, DRS-2, MMSE). The severity of frontal dysfunction, as assessed by FAB was similar in DLB and AD patients. All DLB patients had VH (as for inclusion criteria), whereas none of the AD patients and controls had VH. Parkinsonism signs were present in all DLB patients while none of the AD patients and controls showed extrapyramidal signs. RBD were observed in 16 DLB patients but no in AD patients and controls. Cognitive fluctuations were present only in DLB patients. All DLB patients presented with an abnormal EEG pattern profile consistent with a diagnosis of DLB. None of the AD patients or controls had EEG abnormalities.

### Cortical Thickness

**Figure 1:**
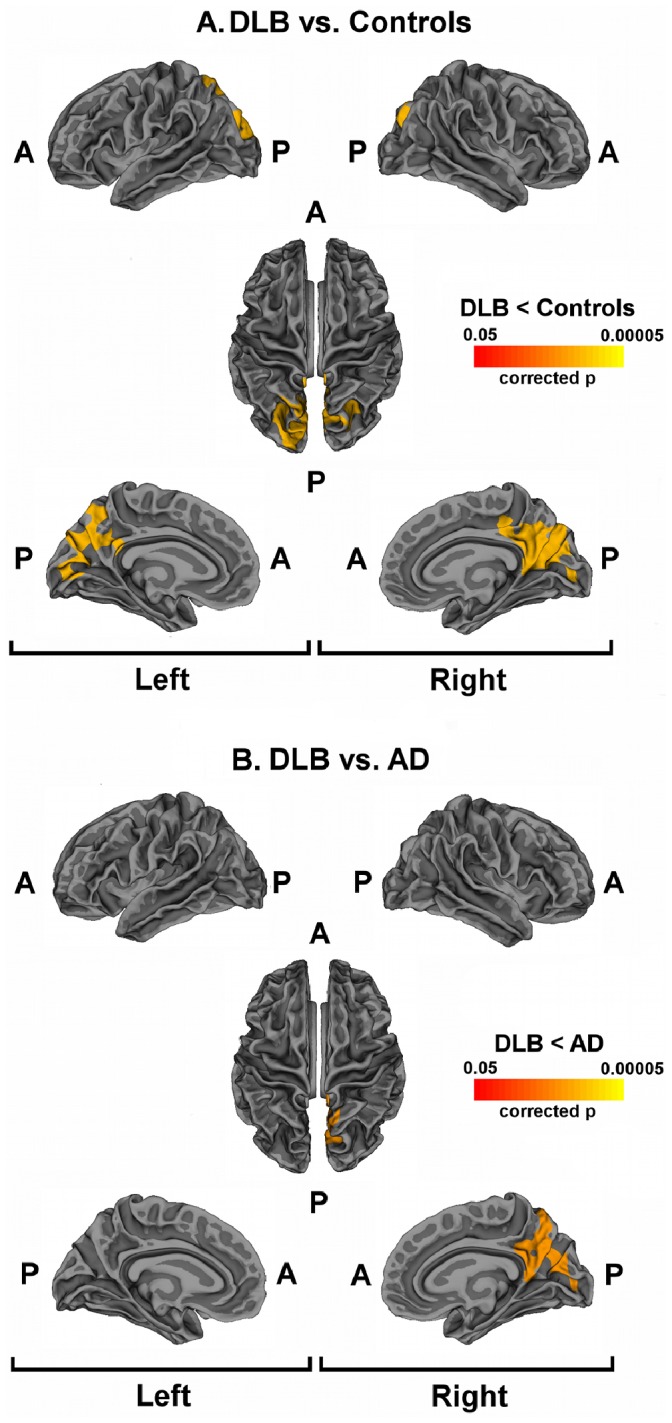
Cortical thinning in DLB patients. Statistical maps showing significant difference between DLB patients and controls (panel A) and between DLB patients and AD (panel B). Areas with cluster wise probability below p = 0.05 are shown, according to Monte Carlo correction for multiple comparisons. Panel A: Colour rating from red to yellow indicates local cortical thickness reduction in DLB patients compared to controls. Panel B: Colour rating from red to yellow indicates local cortical thickness reduction in DLB patients compared to AD. Abbreviations: A = Anterior; P = Posterior.

**Figure 1** **panel A** shows regional differences (corrected p<0.05) in cortical thickness between DLB and age-matched healthy controls. Cortical thickness was reduced in the posterior regions of DLB. Specifically, pericalcarine, lingual gyri, cuneus precuneus and superior parietal gyrus were affected bilaterally.

**Figure 1** **panel B** shows regional differences (corrected p<0.05) in cortical thickness between DLB and AD. Cortical thickness was reduced in DLB compared to AD in the right posterior regions including superior parietal gyrus, precuneus, cuneus, pericalcarine and lingual gyri.

**Figure 2:**
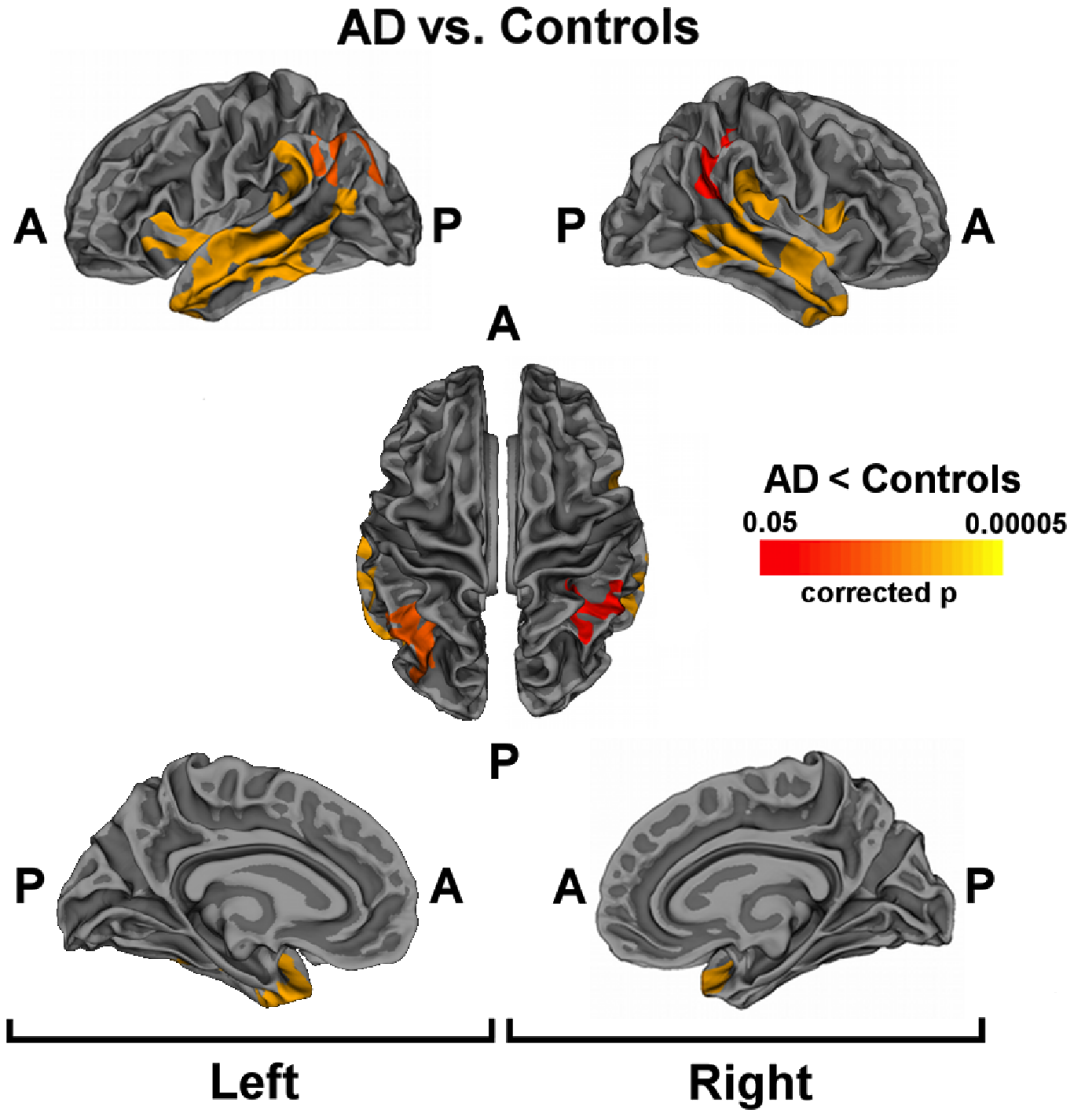
Cortical thinning in AD patients. Statistical maps showing significant differences between AD patients and controls. Areas with cluster wise probability greater than p = 0.05 are shown, according to Monte Carlo correction for multiple comparisons. Colour rating from red to yellow indicates local cortical thickness reduction in AD patients compared to controls. Abbreviations: A = Anterior; P = Posterior.

**Figure 2** shows regional differences (corrected p<0.05) in cortical thickness between AD patients and control subjects. In AD cortical thinning was found bilaterally in temporal cortex, inferior parietal and supramarginal gyri. Temporal regions were widely affected in AD patients in both hemispheres, particularly in the left hemisphere. Specifically, cortical thinning was observed in the superior and middle temporal gyrus, part of inferior temporal cortex, temporal pole and insula.

### Correlation Analysis

**Figure 3:**
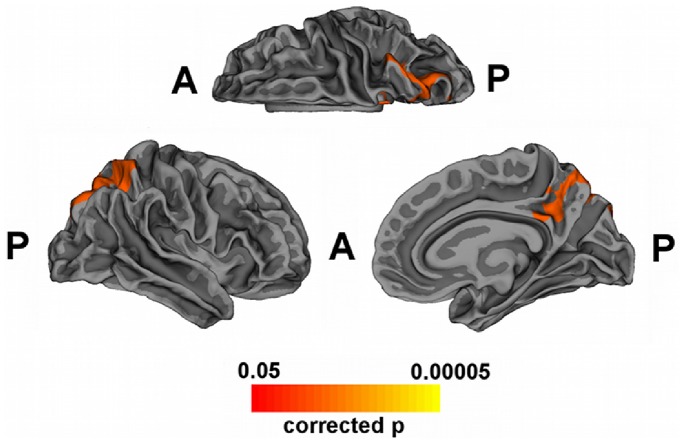
Correlation between cortical thickness and NPI hallucination item scores in DLB patients. Statistical map shows significant regions where the cortical thinning in DLB patients and the NPI hallucination item were correlated (corrected p<0.05). Colour rating from red to yellow indicates local negative correlation.

**Figure 3** shows cortical regions where cortical thickness and NPI hallucination item scores were significantly correlated (corrected p<0.05) in DLB group. These regions were located in the right hemisphere and they included the precuneus and superior parietal gyrus. **Table 2** reports partial correlations between NPI hallucination item scores and cortical thinning values in posterior regions (superior parietal gyrus, precuneus, cuneus, pericalcarine and lingual gyri) which were more altered in DLB than in AD.

**Table 2:** Partials correlations between NPI hallucination item scores and cortical thinning values in posterior regions which were significantly affected in DLB respect to AD.

Cortical region	p	R
R-cuneus	0.834	−0.059
R-lingual	0.544	−0.166
R-pericalcarine	0.532	−0.175
R-precuneus	**0.037**	**−0.543**
R-SP cortex	**0.028**	**−0.565**
L-cuneus	0.364	−0.253
L-lingual	0.623	−0.138
L-pericalcarine	0.363	−0.253
L-precuneus	0.051	−0.512
L-SP cortex	0.072	−0.478

Bold characters indicate statistically significant results after post-hoc analysis.

a MMSE, CAF, UPDRS were added to model as nuisance factors;

b df = 13; R = right; L = left; SP = superior parietal.

## Discussion

With the present MRI study, we have evidenced that DLB patients are characterized by marked cortical thinning in posterior regions when compared with AD and age-matched healthy controls.

Posterior cortical regions play a critical role in the visual information processing, and their damage was related to recurrent VH in DLB patients [6], [23], [24].

Consistently with neuroimaging [25]–[27], neuropathological [4], [28], [29] and electrophysiological [30], [31] studies, we found structural abnormalities in the cuneus and in the higher visual areas related to VH in DLB group.

Although previous studies reported abnormalities related to VH in DLB or Parkinson’s Disease (PD) patients compared to control groups [25]–[26], our findings reveal that the posterior cortical regions were clearly thinned in the comparison between DLB and control subjects, but also in the comparison between DLB and AD patients evidencing that the changes are not linked to a generic neurodegenerative (dementia) process. However, this observation is limited only to DLB and AD. Indeed, previous studies reported an association between cortical thinning in posterior areas and disease stages and cognitive deterioration in Parkinson’s disease patients [32]–[34], who share several clinical commonalities with DLB patients. Future studies could evaluate if the structural cortical alterations could be related to the presence of VH occurring in the later stages of PD.

Additionally, despite both the dorsal and ventral attention networks were affected in DLB as compared to controls and AD, the correlation analysis between cortical thickness and NPI hallucination item scores, suggested a greater involvement of the dorsal attention regions including superior parietal gyrus and precuneus in the occurrence and severity of VH in DLB.

The superior parietal region is implicated in visuo-spatial working memory [35], retrieval and construction of spatial representations [36], processing of visuospatial tasks [37] and filtering distracting information [38]. In this way, deficits in visual attention and in dorsal attention network have been described as an important factor in the causal models of VH [39], [40]. Particularly, we hypothesize that the reduction of cortical thickness in the superior parietal regions may lead to the disruption in the visual attention pathways, leading to the incorporation of stereotyped form-objects into the visual field and predisposing to VH [40].

Precuneus acts in concert with the parietal regions, elaborating information about motor imagery and more abstract mental imagery tasks [41]. Furthermore, precuneus activity has been related to visual perseveration, autoscopic phenomena and visual perseveration or recurrent appearance of a visual image after the stimulus has disappeared [2]. In agreement with our findings, a VBM study has demonstrated the relationship between precuneus atrophy and the presence of VH in DLB patients [6]. PET studies on DLB with VH reported hypoperfusion and hypometabolism in occipital cortex (Brodmann area 17–19) and in precuneus [42]–[43] as compared to control subjects. Posterior region of precuneus has been described as a critical node of information convergence in the parietal network [41] and its damage strongly supports the hypothesis of impaired dorsal visual processing in DLB patients [39].

Despite impairment of dorsal stream is well defined in DLB patients, the ventral stream resulted to be affected in both AD and DLB as compared to controls. However a different distribution of grey matter loss along the ventral pathway was found in DLB and AD. Specifically, cortical thinning in lingual and pericalcarine giri was observed in DLB, whereas a marked and diffuse cortical thinning was found in the mesial temporal cortex in AD. This latter structural alteration could be linked to greater memory impairment affecting AD patients and it is in accordance with previous morphometric studies [9], [44], [45]. Cortical thinning in ventral stream of DLB could be related to greater dysfunction in visual perception [2].

According to previous reports from our group [46]–[47], in the current study we observed a higher prevalence of cortical thinning in the right hemisphere in DLB patients. These findings might suggest a specific role of the right hemisphere in the occurrence of VH in DLB patients. A possible interpretation of these findings could be given on the basis of established and dominant role of the right hemisphere in visuo-spatial attention [48]. However, further investigations across both functional and structural techniques are required to clarify whether right hemispheric dysfunction is truly a specific feature of DLB.

Different models have suggested a central role of posterior regions in manifestation of VH [39], [49]. Recently, Shine et al. [40] have hypothesized that in presence of inability to activate the dorsal attention network, the visuo-perceptual errors are processed by neuronal networks unprepared for this task, such as the default mode network and the ventral attention network. In this context, our group have recently shown that DLB patients characterized by fluctuations of alertness, RBD and VH may be linked to persistent default-mode network activity [47]. Based on these evidences, we proposed that structural changes in key regions pertaining to dorsal attention network may be central to originate VH in DLB patients.
